# Contributory Factors for Teen Insomnia Symptoms: A Prospective Cohort Study in Sweden

**DOI:** 10.3389/fnins.2022.904974

**Published:** 2022-06-28

**Authors:** Gita Hedin, Annika Norell-Clarke, Hanne Tønnesen, Albert Westergren, Pernilla Garmy

**Affiliations:** ^1^Faculty of Health Sciences, Kristianstad University, Kristianstad, Sweden; ^2^Clinical Health Promotion Centre, WHO-Collaborating Centre, Department of Health Sciences, Faculty of Medicine, Lund University, Lund, Sweden; ^3^Department of Social and Psychological Studies, Karlstad University, Karlstad, Sweden

**Keywords:** adolescents, children, family affluence, insomnia, longitudinal study, sleep duration

## Abstract

**Objectives:**

Insufficient sleep is a public health problem that impacts the mental and physical health of children and adolescents. Complaints of insomnia are particularly pervasive among adolescents. This longitudinal study investigates factors that contribute to teen insomnia symptoms.

**Design:**

Five-year prospective follow-up study.

**Setting:**

School-based.

**Participants:**

A total of 522 children (49.8% girls) aged 9.4 ± 1.3 years at baseline; 14.4 ± 0.7 years at follow-up.

**Measurements:**

The dependent variable of insomnia symptoms at follow-up was assessed with the Minimal Insomnia Symptom Scale-Revised. The independent variables at baseline were the perceived family financial situation, tiredness at school, problems waking up, short sleep duration, sleeping difficulties, having a bedroom Television (TV), and time spent with a TV/computer. Multivariate binary logistic regression analyses were used to examine whether the independent variables at baseline predicted insomnia symptoms at follow-up.

**Results:**

Perceived quite bad/very bad family financial situation (OR 3.1; CI 1.4–6.7) and short sleep duration (<10 h) (OR 2.3; CI 1.0–5.3) among girls at baseline were associated with insomnia symptoms at follow-up. Having problems waking up among boys at baseline was associated with insomnia symptoms at follow-up (OR 4.9; CI 1.6–14.4).

**Conclusion:**

Short sleep duration, problems waking up, and perceived bad family financial situation during childhood were linked with adolescent insomnia symptoms. The sex-based differences in these associations warrant further investigation to effectively mitigate adolescent insomnia.

## Introduction

Short sleep duration, insomnia symptoms, and daytime sleepiness are common among adolescents (Shochat et al., 2014). Sleep problems in childhood and adolescence increase the risk for emotional and behavioral difficulties in adulthood (Gregory and Sadeh, 2012; Shochat et al., 2014), so it is important to identify factors in childhood that are predictive of insomnia symptoms in adolescence. There are factors over which adolescents have no influence but may impact their sleep, such as their sex and their family’s financial situation. Previous studies found that insomnia after puberty is more common among females than males (Zeng et al., 2020). Also, adolescents in families with downward socioeconomic mobility were more likely to report sleep problems (Sivertsen et al., 2017). As such, it is vital to consider sex and family affluence when investigating adolescent sleep. Also, biological development processes such as puberty affect sleep and sleep patterns among adolescents (Carskadon, 2011). For example, adolescents can change from being alert in the morning to becoming alert in the evening (Colrain and Baker, 2011). Even though this nocturnal shift is an expected aspect of this developmental stage, late evening habits and early morning school-start times cause many adolescents to have insufficient sleep (Colrain and Baker, 2011).

Sleep duration changes through life due to biological and social factors connected to growing and aging (Bódizs, 2021). Thus, the recommended sleep duration varies according to age and context. Sufficient sleep is defined as the amount of sleep required for optimal daytime functioning (Reynolds and Banks, 2010). Recommendations from the National Sleep Foundation in the United States for nightly sleep duration are 9–11 h for school-aged children (6–13 years old) and 8–10 h for teenagers (14–17 years old) (Hirshkowitz et al., 2015). These recommendations are close to those in Sweden (10–11 h for children aged 6–12 and 8–9 h for adolescents aged 13–18), which might reflect national sleep habits (Andree et al., 2015). A cross-sectional study among adolescents (13–15 years old) shows that 31% receive less than 8 h of sleep on weekdays (Garmy et al., 2020). Insufficient sleep is a pervasive and prominent public health concern that is being exacerbated by modern “24/7” society (Chattu et al., 2018). Not having enough sleep is associated with adverse physical health outcomes, poor mental health, and attention and behavior problems. Thus, understanding and addressing this growing worldwide issue is of the utmost importance (Johnson et al., 2018; Bruni et al., 2019).

The most common sleep disorder, insomnia, involves difficulties initiating and maintaining sleep or waking up too early to the extent that it disrupts functioning on the next day (American Psychiatric Association, 2013). Recent versions of the ICD-10 (World Health Organization, 2010) and DSM-5 (American Psychiatric Association, 2013) diagnostic manuals emphasize affected daytime functioning as a diagnostic criterion for insomnia, which was not stressed to the same extent in earlier versions (Ellis, 2021). The Minimal Insomnia Symptoms Scale (MISS) (Broman et al., 2008) is a screening instrument that has recently been revised (MISS-R) to include an item about daytime functioning (Hedin et al., 2022). The MISS-R has been found to be reliable and valid among adolescents (Hedin et al., 2022).

The trajectory of insomnia in childhood and adolescence is not fully understood. Earlier studies suggest a stability of childhood insomnia, albeit with an increasing trend from childhood to adolescence (Zhang et al., 2011; Falch-Madsen et al., 2020). In a longitudinal study in Norway of a 10-year follow-up from age 4 to 14, there was an increase in insomnia rates from 2.5% at the age of 4–6 years to 7.5–12.3% at the ages of 8–14 years (Falch-Madsen et al., 2020). A longitudinal study from Hong Kong found that the prevalence of insomnia increased from 4.2% at baseline (children aged 9 years) to 6.6% at follow-up when the adolescents were 14 years old (Zhang et al., 2011).

Lacking or altered sleep can disrupt daytime functioning, meaning decreased cognitive abilities and increased difficulties in regulating and expressing emotions. In a meta-analysis, adolescents with insomnia were found to have an increased risk of reporting depressive symptoms later (at least 12 months to evaluate the long-term relationship between insomnia and depression) (Baglioni et al., 2011). Also, both academic stress and family and peer-related conflicts were associated with insomnia in adolescence (Bernert et al., 2007).

After puberty, women are more likely to report experiencing insomnia than men, and the reasons for this are not fully understood (Zhang and Wing, 2006). Sleep differences between males and females may be due to differences in sleep-regulating hormones (Zeng et al., 2020), and social factors may also partially underlie these sex-based differences (Chen et al., 2005). Knowledge and awareness gaps exist concerning the sleep differences between sexes (Mallampalli and Carter, 2014), both of which warrant further investigation. In one study describing sex-based differences in sleep patterns and insomnia among 16–18-year-olds (Hysing et al., 2013), female adolescents reported a higher rate of insomnia than males, while males reported later bedtimes and greater weekday–weekend discrepancies (Hysing et al., 2013). However, Norell-Clarke and Hagquist (2017) showed that 15-year-old males were more likely than females to have late bedtimes and to sleep less than recommended. As such, it is crucial to take sex into account when investigating sleep.

A longitudinal study among 252 adolescents (aged 16) showed that perceived disadvantaged financial situations relate to having less sleep than recommended (Philbrook et al., 2020). A disadvantaged financial situation (objectively or subjectively) or living in an insecure residential area has negatively impacts on sleep, such as shorter sleep duration and reduced sleep quality (Jarrin et al., 2014; Troxel et al., 2020). Another aspect of socioeconomic status that affects sleep relates to career ambitions. Aiming for a vocational track rather than a higher-educational track is associated with late bedtimes and less sleep duration in teenagers (Norell-Clarke and Hagquist, 2017). Thus, socioeconomic factors are important to consider when investigating adolescent sleep.

One of the benefits of longitudinal studies is that they provide an overview of phenomena over time. As such, this study design enables identification of factors in childhood that increase the risk of developing insomnia in adolescence, which may eventually be used to prevent insomnia. Since insomnia can differ between boys and girls, they were investigated separately in this study.

The “Sleep and Lifestyle” instrument was developed for measuring sleep length, television habits, and computer habits of Swedish school-age children and was psychometrically evaluated among children and adolescents aged 6–16 years (Garmy et al., 2012a). In a cross-sectional study (Garmy et al., 2012b), an association was found at baseline between short sleep length and being tired at school, difficulties waking up, difficulties falling asleep, having a bedroom Television (TV), and spending ≥ 2 h with a TV/computer. Follow-up data collected in 2015–2019 included the MISS-R. The purpose of the current study was based on the cross-sectional associations found in an earlier study at baseline (Garmy et al., 2012b). The study investigates whether short sleep length (<10 h), being tired at school, difficulties waking up, difficulties falling asleep, having a bedroom TV, spending ≥ 2 h with a TV/computer, and perceived family financial situation among 9-year-old boys and girls predict insomnia symptoms as adolescents at the age of 14.

## Materials and Methods

Data were obtained from the “Sleep and lifestyle in school age children” study (ISRCTN17006300) (Garmy, 2008). A longitudinal design was used in this study. The participants consisted of all children born in 2001 and 2002 in a municipality with rural and urban areas in Southern Sweden (*n* = 2,308). The first round of questionnaires (baseline) was distributed in 2008–2013; and the second round (follow-up) was distributed in 2015–2019. The cohort included 44 schools (both public and private tuition-free schools). At the time of the data collection, about 17% of the population was born abroad. The unemployment rate of the parents was 2.4%. Welfare payments were provided to 1.6% of the population. The education level in the municipality was higher than the Swedish average (i.e., 63% had post-secondary school education). About 82% of 6-year-old children lived with both parents compared with 63% for 17-year-old adolescents (Statistics Sweden, 2019).

**FIGURE 1:**
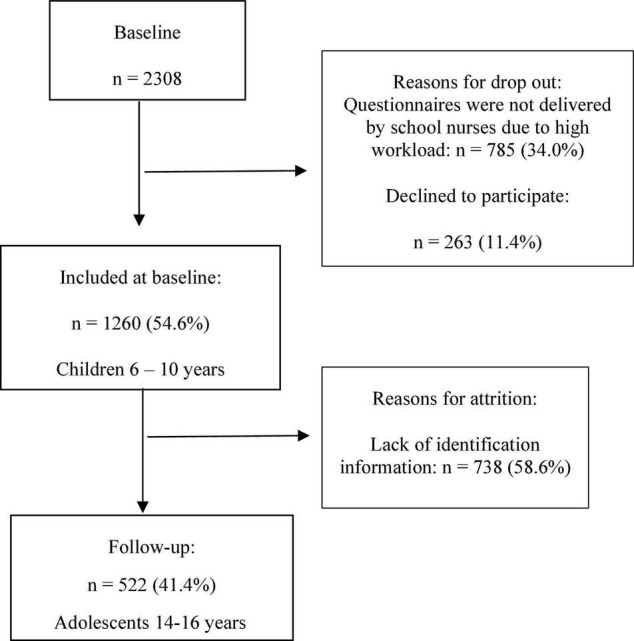
Selection process at baseline and follow-up.

Written information about the purpose of the study and its voluntary nature was distributed to students and their guardians, of whom 1,260 (54.6%) provided their written informed consent to participate. At baseline, the questionnaire was completed by children together with their guardians. At follow-up, the adolescents responded to the questionnaire during school hours (Figure 1). Out of the 1,260 questionnaires completed at baseline, 522 (41.4%) provided identifying information that enabled tracking of the students for the follow-up survey as adolescents.

### Instruments

The MISS-R served as the assessment tool at follow-up. Three questions in the MISS (Broman et al., 2008; Hellström et al., 2010) and MISS-R (Hedin et al., 2022) were in accordance with the criteria of the International Classification of Diseases 10th revision (ICD-10), including the cardinal symptoms of insomnia: difficulty falling asleep, difficulty maintaining sleep, or non-refreshing sleep (Broman et al., 2008; World Health Organization, 2010; American Psychiatric Association, 2013). The third item referring to non-refreshing sleep was revised in the MISS-R to instead assess daytime functioning, which is a diagnostic criterion in the current version of the ICD-10 (World Health Organization, 2010). Each item has five response categories: no, minor, moderate, severe, and very severe problems, which are scored 0–4, respectively. This yields a total score of 0–12, where higher scores indicate more severe insomnia, and a cutoff score of ≥6 is considered indicative of insomnia (Hedin et al., 2022). The MISS-R was psychometrically tested among adolescents and found to have good psychometric properties (Hedin et al., 2022).

The “Sleep and Lifestyle” instrument was distributed at baseline. It has been psychometrically evaluated earlier and was found to be valid and reliable for this age group (Garmy et al., 2012a). The instrument includes questions about being tired in school (4-point Likert scale ranging from “never” to “every day”), problems waking up (4-point Likert scale ranging from “never” to “every morning”), sleep duration the night before school (hours and minutes), difficulties falling asleep (4-point Likert scale ranging from “never” to “every night”), having a bedroom TV (yes/no), and time spent with a TV/computer (hours, minutes). Questions about sex and perceived family financial situation were also included (5-point Likert scale ranging from “very well” to “very bad”).

### Data Analyses

Data were first analyzed among females and males as frequencies (*n*) and percentages (%). Bivariate analyses (chi-squared and Mann–Whitney *U* tests) were used to identify associations between the dependent variable of insomnia symptoms at follow-up (MISS-R; <6 = no insomnia and ≥ 6 = insomnia) and the following independent variables at baseline: tiredness in school (never = 0; seldom = 1; often = 2; every day = 3); waking difficulties (0 = never; 1 = seldom; 2 = often; 3 = every day); sleep duration (0 = ≥10 h; 1 ≤10 h); difficulties falling asleep (never = 0; seldom = 1; often = 2; every night = 3), having a bedroom TV (yes = 1; no = 0) and time spent with TV/computer (<2 h = 0; 2 h = 1) and perceived family financial situation (very good, quite good/average = 0; quite bad/very bad = 1). The cutoff of 10 h for sleep duration was in accordance with the Swedish sleep recommendations for children in this age group (Andree et al., 2015). Since there was a fairly small group of students who reported sleeping less than 9 h/night in this study (14.9%), we chose to follow the Swedish recommendations rather than those of the United States National Sleep Foundation (Hirshkowitz et al., 2015).

Multiple logistic regression analysis was then used to examine the associations between the dependent variable of insomnia symptoms at follow-up (MISS-R ≥ 6) among female and male adolescents and the same independent variables at baseline as in the bivariate analyses. All independent variables were included in a single model without any additional variable selection (“enter” method in SPSS). The rationale for using a logistic regression analysis is that it allows us to see the effects of variables after adjusting for other variables. This helps us verify that the associations seen in the bivariate analysis are not due to the influence of other variables. The independent variables in the logistic regression were dichotomized: tiredness in school (never/seldom = 0; often/every day = 1), difficulties waking up (never/seldom = 0; often/every day = 1), difficulties falling asleep (never/seldom = 0; often/every night = 1), and perceived family financial situation (very good/quite good/average = 0; quite bad/very bad = 1). This was done due to the low response rates in certain response categories. The model fit was assessed with the Hosmer–Lemeshow goodness-of-fit test, which assesses differences between the actual and predicted values of the dependent variable. Good model fit is indicated by a non-significant p-value, indicating no difference between the actual and predicted dependent values (Hair et al., 1998). Multicollinearity was assessed by tolerance, which should be >0.4 (Edlund, 1994). Nagelkerke’s pseudo-*R*^2^ indicated how well the model accounted for the odds of insomnia symptoms according to the MISS-R (Field, 2018). IBM SPSS v. 26 (IBM Corp., Armonk, NY, United States) was used for statistical analyses with the level of significance set at *p* < 0.05.

## Results

Out of the 522 included participants, 49.8% were girls, and 50.2% were boys. The mean age at baseline was 9.43 years (SD 1.28), and the mean age at follow-up was 14.28 years (SD 0.69). The mean time from baseline to follow-up was 4.85 years (SD 1.41). Insomnia symptoms measured with the MISS-R were significantly more common among female adolescents (16.5%) than male adolescents (6.5%; <0.001). The bivariate analysis (Table 1) showed that a quite bad/very bad perceived family financial situation and short sleep duration (<10 h/night during the school week) among girls at baseline were associated with reported insomnia symptoms among female adolescents at follow-up (*p* ≤0.001 and *p* = 0.020, respectively). Difficulty waking up among boys at baseline was associated with insomnia symptoms among male adolescents at follow-up (*p* = 0.007).

**TABLE 1:** Bivariate analysis of associations of insomnia (MISS-R ≥ 6) at follow-up and factors at baseline.

	Adolescent females			Adolescent males		
Factors at baseline	No insomnia (MISS-R < 6)	Insomnia (MISS-R ≥ 6)	*P*	No insomnia (MISS-R < 6)	Insomnia (MISS-R ≥ 6)	*p*
**Perceived family financial situation, *n* (%)**						
Very good/quite good/average	178 (84.4)	26 (60.5)	<0.001^a^	193 (80.1)	11 (64.7)	0.132^a^
Quite bad/very bad	33 (15.6)	17 (39.5)		48 (19.9)	6 (35.3)	
Tired in school, *n* (%)			0.069^b^			0.320^b^
Never	44 (20.9)	8 (19.0)		56 (23.0)	5 (29.4)	
Seldom	154 (73.0)	28 (66.7)		173 (71.2)	10 (58.8)	
Often	12 (5.7)	5 (11.9)		12 (4.9)	2 (11.8)	
Everyday	1 (0.5)	1 (2.4)		2 (0.8)	0 (0.0)	
Difficulties waking up, *n* (%)			0.276^b^			0.007^b^
Never	35 (16.4)	5 (11.6)		72 (29.9)	0 (0.0)	
Seldom	102 (47.9)	19 (44.2)		113 (46.9)	8 (47.1)	
Often	62 (29.1)	13 (30.2)		50 (20.7)	6 (35.3)	
Every morning	14 (6.6)	6 (14.0)		5 (2.5)	3 (17.6)	
Sleep duration,*^c^* *n* (h)			0.020^a^			0.884^a^
≥10 h sleep	101 (47.2)	12 (27.9)		109 (45.2)	8 (47.1)	
<10 h sleep	113 (52.8)	31 (72.1)		132 (54.8)	9 (52.9)	
Difficulties falling asleep			0.246			0.551^a^
Never	62 (29.0)	8 (18.6)		73 (30.0)	3 (17.6)	
Seldom	129 (60.3)	27 (62.8)		143 (58.8)	13 (76.5)	
Often	20 (9.3)	6 (14.0)		25 (10.3)	1 (5.9)	
Every night	3 (1.4)	2 (4.7)		2 (0.8)	0 (0)	
Having a bedroom TV	45 (21.0)	14 (32.6)	0.241	48 (19.8)	5 (29.9)	0.353^a^
Spending 2 h with TV/computer	79 (37.8)	14 (45.2)	0.390	115 (48.3)	9 (52.9)	0.804^a^

*MISS-R, Minimal Insomnia Symptom Scale-Revised-Revised. ^a^Chi-square test. ^b^Mann–Whitney U test. ^c^During school week. Missing: <3.5%.*

The multiple logistic regression analysis (Table 2) showed that perceived quite bad/very bad family financial situations at baseline were associated with insomnia symptoms according to the MISS-R in female adolescents at follow-up (OR 3.1; CI: 1.4–6.7). Short sleep duration (<10 h) at baseline was associated with insomnia symptoms according to the MISS-R in female adolescents at follow-up (OR 2.3; CI: 1.0–5.2). Problems waking up at baseline were linked to insomnia symptoms in male adolescents at follow-up (OR 4.9; CI: 1.7–14.4).

**TABLE 2:** Multiple logistic regression analysis of the associations between insomnia (MISS-R ≥ 6) at follow-up and factors at baseline, *n* = 522.

	Adolescent females (14–16 years)^1^				Adolescent males (14–16 years)^2^			
Factors at baseline	Wald	OR	95% CI for OR	*p*	Wald	OR	95% CI for OR	*p*
Poor family financial situation*	7.97	3.06	1.41–6.67	0.005	1.80	2.12	0.71–6.39	0.179
Often tired at school	0.83	1.71	0.54–5.47	0.362	1.01	2.43	0.43–13.71	0.314
Often difficulty waking up	0.32	1.24	0.59–2.62	0.569	8.21	4.87	1.65–14.41	0.004
Less than 10 h sleep^3^	4.12	2.32	1.03–5.25	0.042	0.17	0.79	0.26–2.39	0.679
Often difficulties falling asleep	1.37	1.81	0.67–4.88	0.242	1.70	0.23	0.02–2.10	0.193
Having bedroom TV	3.76	2.22	0.99–4.97	0.052	1.26	1.96	0.60–6.32	0.262
Spending 2hr with TV/computer	0.14	1.15	0.56–2.39	0.704	0.55	1.52	0.50–4.66	0.458

*, bad/very bad family financial situation. CI, confidence interval; OR, odds ratio. ^1^Hosmer–Lemeshoow goodness-of-fit test, p = 0.520; Naglekerke’s pseudo-R^2^ = 0.148. ^2^Hosmer–Lemeshoow goodness-of-fit test, p = 0.644; Naglekerke’s pseudo-R^2^ = 0.127; there were no signs of multicollinearity (tolerance: 0.7–0.8). ^3^Less than 10 h of sleep during school week.

## Discussion

The aim of this longitudinal study was to investigate which factors can predict insomnia in female and male adolescents. The findings revealed that perceived quite bad/very bad family financial situations among girls at baseline were associated with insomnia symptoms at follow-up. The data also indicated that short sleep duration among girls at baseline increased the risk of developing insomnia symptoms in female adolescents. A third statistically significant finding was that difficulties waking up in boys at baseline was associated with insomnia symptoms at follow-up. However, tiredness in school, difficulties falling asleep, electronic media use (bedroom TV and time spent with a TV/computer) were not significant in female and male adolescents. This was surprising since our earlier cross-sectional study had found associations between insufficient sleep and these variables at baseline. However, other longitudinal studies from Norway (Falch-Madsen et al., 2020) and Hong Kong (Zhang et al., 2011) show that the trajectory for childhood insomnia is not fully understood, and further studies are needed.

Previous studies report that adolescents with insomnia symptoms also have poor attention, difficulty concentrating, academic frustration, poor interest, and low motivation levels in school (Zhao et al., 2019). However, it is important to consider the contextual factors beyond adolescents’ control that affect their sleep. An international investigation reports that socioeconomic differences affect young people’s self-reported health when they grow up (WHO, 2013/2014). Family and peers also factor into the context of sleep among adolescents (Becker et al., 2015). Among females in this study, there were higher odds of insomnia symptoms when they perceived their family financial situation to be quite bad/very bad. Girls’ greater sensitivity to the family financial situation may have to do with gender-specific norms, behaviors, and roles (World Health Organization, 2022).

This finding is supported by a systematic review (Felden et al., 2015) that also identified an association between socioeconomic level and sleep in adolescents. Low socioeconomic status is associated with a worse subjective perception of sleep quality, sleep problems/disturbance (i.e., insomnia), shorter sleep duration, and more daytime sleepiness (Felden et al., 2015). This is not yet clearly understood, but other studies show associations between disadvantaged financial situations/insecure residency, short sleep duration, and poor sleep quality (Jarrin et al., 2014; Troxel et al., 2020). The findings from a repeated cross-sectional study evidence associations between sleep disturbance, being female, and worry over one’s family financial situation (Danielsson et al., 2016).

Our study is in line with previous findings that female adolescents have more insomnia symptoms than male adolescents (Jakobsson et al., 2019). Our results indicate that short sleep duration among girls predicted insomnia in female adolescents. Although insufficient sleep was self-reported by 54% of the boys and 56% of the girls at baseline, insufficient sleep was only predictive of later developing insomnia symptoms among the girls.

The causes of short sleep duration in our study are not known. However, worries such as school demands have been reported to be linked with insomnia symptoms beyond short sleep duration (Leach and Hubert, 2021). It is possible that boys and girls have insufficient sleep for different reasons, such as voluntarily or involuntarily lack of sleep. Furthermore, our study supports previous observations of difficulties waking up on school days being common during adolescence (Leach and Hubert, 2021). However, only boys with difficulties waking up in their younger years had an increased risk of insomnia symptoms at follow-up.

It should be noted that symptoms of insomnia are prominent in adolescents with delayed sleep-wake phase disorder (DSWPD) (Gradisar et al., 2011) and that difficulties waking up might be an early symptom of DSWPD rather than insomnia. The prevalence of DSWPD during adolescence is likely underrated (Gradisar et al., 2011). DSWPD is often misdiagnosed as insomnia because both involve difficulties falling asleep in the evening, but those with DSWPD succeed in attaining sufficient sleep if they are allowed to sleep according to their circadian rhythms (Zucconi and Ferri, 2021).

The results of the present study add to the knowledge on factors that predict insomnia among adolescents. Recommendations for school healthcare include screening for factors that predict insomnia symptoms. Difficulties waking up in the morning should alert school health professionals to enquire further about boys’ sleep. Also, insecure financial situations should warrant concern regarding insomnia symptoms as well, especially among girls. Screening with the MISS-R could be a starting point for these kinds of discussions. Effectively supporting healthy sleep behavior in adolescence requires an understanding of why girls with short sleep durations are more at risk of developing insomnia than boys and why boys are more affected than girls in terms of difficulties waking up.

### Strengths and Limitations

One strength of the current study is its longitudinal design, which can yield greater knowledge by following the same individuals over time and establishing a timeline. The large sample size is also a strength, although the relatively high attrition due to difficulties identifying students from baseline to follow-up is a limitation. We did not enquire about the reasons underlying attrition, but the sex ratio, mean age, sleep duration, and perceived family financial situation did not differ between participants who completed both questionnaires and those who participated at the first time point only. We do not know whether there were other differences between participants and non-participants that could have affected the investigated variables.

Other study limitations include aspects that were not investigated in this study, such as whether the students’ parents live together or whether they reside in a single-parent household, as well as the children’s general health, insomnia at baseline, and the family’s objective financial situation. Another limitation is that no objective measurements were used. At baseline, the children and their guardians answered the survey questions together due to the children’s young age. Although this allowed us to gather data from young school children who otherwise may not have been capable of self-reporting on certain topics (e.g., family affluence), it might have elicited different answers compared with the later time point when the adolescent children responded to the questionnaire without their guardians present. The items in the logistical regression were dichotomized due to the low response rate for some options. The lack of an insomnia measure at baseline is also a limitation, rendering it impossible to control for the effects of childhood insomnia on adolescent insomnia. However, difficulties of falling asleep at baseline were not associated with insomnia symptoms at follow-up.

The sensitivity and specificity of MISS-R in comparison to a gold standard remains to be explored. We are aware of the complexity of sleep and that it could be valuable to explore more non-sleep exposure. However, at the time of data collection at baseline in the 2008–2013, some of the challenges with high use of smartphones and nighttime texting were not as present as they are today. Also, the surveys were distributed at regular school health visits, so there was also a time aspect that limited the numbers of questions so as not to take too much time from the health visit.

Having less than 10 h of sleep and the family financial situation among girls at baseline contributed to insomnia in adolescent females at follow-up. Problems waking up among boys at baseline contributed to insomnia in adolescent males at follow-up. The sex-based differences in these associations require further investigation to understand adolescent insomnia.

## Data Availability Statement

The original contributions presented in this study are included in the article/supplementary material, further inquiries can be directed to the corresponding author.

## Ethics Statement

The study was reviewed by the Advisory Committee on Research Ethics in Health Education at Lund University (VEN 34-09) and Regional Ethical Review Board in Lund (EPN 2011/333, EPN 2015/113, and EPN 2017/600). Written informed consent to participate in this study was provided by the participants or their legal guardian/next of kin.

## Author Contributions

GH, PG, AN-C, AW, and HT: study design. GH and PG: data analysis. GH: writing the first draft of manuscript. GH, PG, AN-C, and AW: manuscript revision. All authors contributed to the work, approved the final version of the manuscript, and agreed with its submission.

## Conflict of Interest

The authors declare that the research was conducted in the absence of any commercial or financial relationships that could be construed as a potential conflict of interest.

## Publisher’s Note

All claims expressed in this article are solely those of the authors and do not necessarily represent those of their affiliated organizations, or those of the publisher, the editors and the reviewers. Any product that may be evaluated in this article, or claim that may be made by its manufacturer, is not guaranteed or endorsed by the publisher.

## Abbreviations

CI: confidence interval
DSWPD: delayed sleep-wake phase disorder
ICD-10: International Classification of Diseases 10th revision
MISS-R: Minimal Insomnia Symptom Scale-Revised
OR: odds ratio
SD: standard deviation.
